# Impact of Exercise on Immunometabolism in Multiple Sclerosis

**DOI:** 10.3390/jcm9093038

**Published:** 2020-09-21

**Authors:** Remsha Afzal, Jennifer K Dowling, Claire E McCoy

**Affiliations:** School of Pharmacy and Biomolecular Sciences, Royal College of Surgeons in Ireland, 123 St. Stephen’s Green, D02 YN77 Dublin, Ireland; remshaafzal@rcsi.com (R.A.); jenniferdowling@rcsi.com (J.K.D.)

**Keywords:** multiple sclerosis, EAE, CNS, exercise, immunometabolism, glycolysis, oxidative phosphorylation, fatty acid metabolism, mitochondria, amino acid

## Abstract

Multiple Sclerosis (MS) is a chronic, autoimmune condition characterized by demyelinating lesions and axonal degradation. Even though the cause of MS is heterogeneous, it is known that peripheral immune invasion in the central nervous system (CNS) drives pathology at least in the most common form of MS, relapse-remitting MS (RRMS). The more progressive forms’ mechanisms of action remain more elusive yet an innate immune dysfunction combined with neurodegeneration are likely drivers. Recently, increasing studies have focused on the influence of metabolism in regulating immune cell function. In this regard, exercise has long been known to regulate metabolism, and has emerged as a promising therapy for management of autoimmune disorders. Hence, in this review, we inspect the role of key immunometabolic pathways specifically dysregulated in MS and highlight potential therapeutic benefits of exercise in modulating those pathways to harness an anti-inflammatory state. Finally, we touch upon current challenges and future directions for the field of exercise and immunometabolism in MS.

## 1. Introduction

### 1.1. Immunopathology of Multiple Sclerosis

Multiple Sclerosis (MS), a debilitating autoimmune disorder of the central nervous system (CNS), has a diverse clinical presentation that comprises of sensory and visual impairments, motor deficits, extreme fatigue, pain and cognitive disturbances. The variations in clinical symptoms associates with the spatiotemporal distribution of pathological lesions within the CNS, a defining feature of MS [1,2,3].

**Figure 1:**
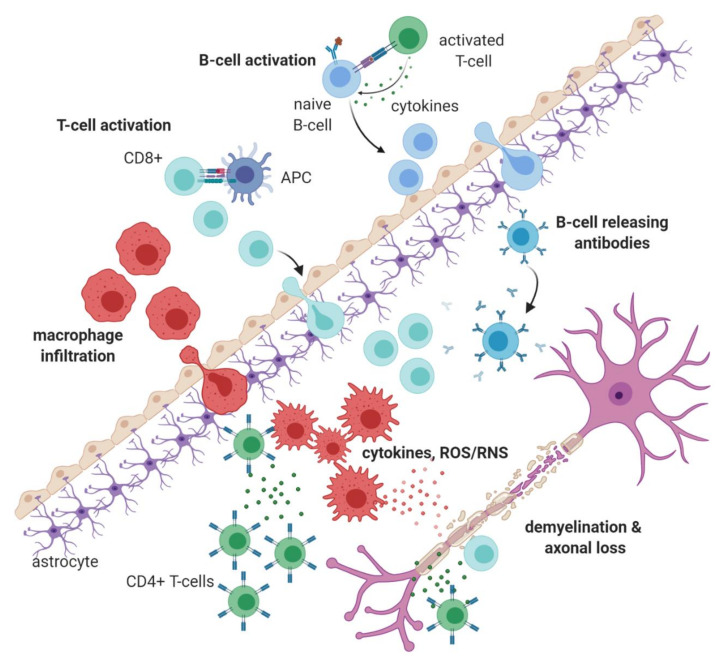
**An outline of MS immunopathology:** MS results from breakdown of the blood-brain barrier (BBB) leading to migration of immune cells into the central nervous system (CNS). These immune cells secrete a range of pro-inflammatory cytokines as well as reactive oxygen and nitrogen species (ROS/RNS), which induce inflammation, formation of sclerotic plaques (lesions), demyelination and axonal degradation. Pathological immune cells in MS include innate immune cells like activated macrophages and resident microglia, autoreactive T-cells (that are activated at peripheral sites, potentially through molecular mimicry, bystander activation or the co-expression of T-cell receptors (TCRs) with different specificities [2]) and antibody producing B-cells. (APC: Antigen-presenting cell); Image made in Biorender^TM^ (BioRender.com).

The most common form of MS is relapsing-remitting multiple sclerosis (RRMS) (affecting about 85% of patients) where peripheral activated immune cells abnormally invade the CNS, leading to inflammatory attacks on myelin sheaths resulting in demyelination. Following demyelination, bare axons are more susceptible to further injury induced by immune cell-derived proinflammatory cytokines and chemokines, like tumour necrosis factor (TNF), interferon-γ (IFN-γ), interleukin-6 (IL-6), interleukin 1 (IL-1), C-C chemokine receptor type 2 (CCR2), and interleukin-17 (IL-17) [1,2,4]. Furthermore, axonal degradation can also be instigated by reactive oxygen and nitrogen species (ROS/RNS) released mainly by infiltrating peripheral macrophages (Figure 1) [1,2,4,5,6,7]. Analysis of lesions in the early stages of MS show proinflammatory ‘M1-like’ macrophages dominating the infiltrate, followed by CD8+ T cells, the numbers of which closely correlate with axonal damage [1,2,8]. Furthermore, autoreactive CD4+ T cell subsets are also found in such lesions (T_H_1 and T_H_17 cells), followed by autoreactive B cells [2,8]. One theory for the emergence and action of such autoreactive B cells and T cells in MS has been attributed to the defective function of regulatory T cells, T_regs_, such as Forkhead Box P3 (FOXP3)-expressing regulatory T cells and IL-10-producing regulatory T cells [2,9]. As the disease progresses, microglial and astrocytic activation are also manifest in the CNS, resulting in heightened grey and white matter atrophy [2,8].

One to two decades post-diagnosis approximately 10–20% of patients with RRMS end up developing secondary progressive multiple sclerosis (SPMS) [10]. In SPMS, inflammatory lesions are no longer typical and instead progressive neurological decline accompanied by CNS atrophy is a more characteristic feature [2,3,6,8]. The third type of MS which develops in 10–15% of patients is the primary progressive form (PPMS), which is characterized by an absence of any relapses and progressive decline from the onset of disease [2,11]. The mechanistic processes that lead to SPMS and PPMS damage are still not fully elucidated, but are thought to be a combination of accrued inflammatory burden and neurodegeneration [2,6,11]. While existing therapies provide some relief to those with RRMS, treatment responses in many RRMS patients remain inadequate. Meanwhile the progressive cases of MS have even fewer treatment options (ocrelizumab for PPMS, and siponimod for SPMS). Hence, it is necessary to build and improve upon other approaches that prevent progression of disease and assist in recovery of neurological function [12,13]. Research over the last decade has shown with increasing evidence that exercise may at least partly fulfil this requirement.

### 1.2. Exercise in Multiple Sclerosis

Research into how exercise (defined as planned, structured and repetitive physical activity that improves fitness [14,15]) affects immune responses, specifically in the fields of anti-tumoral and anti-infection immunity, resulted in it being viewed as a potential therapy for MS [16]. Exercise has shown to improve the well-being of MS patients by enhancing motor function, decreasing fatigue, and improving cardiorespiratory fitness [17,18,19,20].

The murine model of MS, experimental autoimmune encephalomyelitis (EAE), was instrumental in learning the mechanisms via which exercise induces a more positive disease course outcome and decline in clinical symptoms [21,22,23]. At the level of the immune system, it was shown to result in a significant elevation in anti-inflammatory T_regs_, decreased immunoglobulin secretion as well as decreased proinflammatory T_H_1 and T_H_17 production [24]. Moreover, other reports revealed that exercise promoted the release of paracrine IL-6 from muscles and induced an anti-inflammatory response via IL-10 secretion and IL-1β inhibition [25,26]. Moreover, other benefits of exercise on EAE pathology have also been reported: One study showed exercise reduced oxidative stress, a source of endothelial injury, which eventually leads to tight junction dysfunction and loss of blood-brain barrier (BBB) integrity [27]. It was also reported to be protective against memory loss, which has largely been attributed to demyelination of the hippocampus— EAE mice that were treadmill exercised, five times weekly for a month had a better performance on step-down avoidance tasks, employed to test memory ability [28]. Additionally, the exercised group showed a reduction in demyelination with an associated increase in brain-derived neurotrophic factor (BDNF), a protein vital for neurogenesis [19,28].

Generally, modern society has reached a stage characterized by high fat diets and exercise deficiency due to automation, which has led to concomitant increases in lifestyle-related diseases such as obesity, diabetes mellitus, osteoporosis or cardiovascular complications. It comes as no surprise then that MS patients, due to motor deficits, are less physically active than healthy controls and suffer more from those diseases as secondary pathologies [20]. However, one study reported obesity itself is a risk factor for developing MS, with obesity in early life associated with a two-fold increase in risk in both men and women [3]. Similarly, a study on the association between female MS patients and exercise reported that of the subjects who developed MS after the baseline physical activity assessment, there was a pointedly higher percentage of MS cases in the group of women reporting lower physical activity [29]. Additionally, leptin (an adipokine known to be increased in obesity) was found to be increased in the serum and cerebrospinal fluid (CSF) of MS patients. Increased leptin levels were associated with increased inflammatory cytokine secretion. Moreover, there was an increased expression of the Leptin receptor, LepR, on the surface of CD8+ T cells from RRMS patients in relapse, relative to patients in remission or healthy controls [30,31]. Consistent with these findings, a study by Mokhtarzade et al. demonstrated that in women with MS, weight loss following exercise was associated with a decrease in leptin and TNF levels, and an increase in the level of anti-inflammatory cytokine IL-10 as well as the weight-loss associated hormone, adiponectin [32].

Collectively, most of the research conducted so far has demonstrated the positive influence of exercise on MS disease course [25,32,33,34,35]. Although studies have reported heterogeneous results on factors like cytokine levels [25], no study has showed worsening of clinical disease secondary to controlled exercise, and therefore, the consensus appears to be that it should be recommended in patients with MS as an adjuvant therapy [17,19]. However, one caveat in exercise research in MS is that almost all focus has been on the outcomes of exercise training on MS symptoms and quality of life, whereas little research on MS has focused on the metabolic mechanisms underlying the effects of exercise training in immune cells.

## 2. Exercise as a Regulator of Immunometabolism

### 2.1. Overview

**Figure 2:**
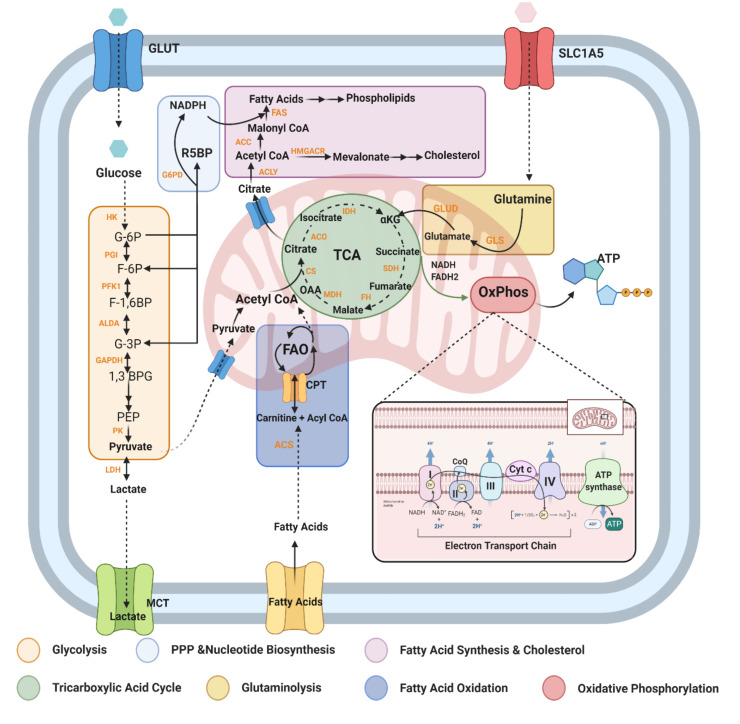
**An outline of key metabolic pathways altered upon immune cell activation:** Cells modify specific metabolic pathways depending on their requirements for activation, growth, development or survival. This capability to shift utilisation of various pathways to generate energy from nutrients like carbohydrates (e.g., glucose), proteins and fats is termed immunometabolism. ATP: Adenosine Triphosphate; LDH: Lactate Dehydrogenase; MCT: Monocarboxylate Transporter; HK: Hexokinase; G-6P: Glucose-6-Phosphate; PGI: Phosphoglucoisomerase; F-6P: Fructose-6-phosphate; PFK1: Phosphofructokinase 1; F-1,6 BP: Fructose 1,6-Bisphosphate; ALDA Aldolase; G-3P: Glyceraldehyde-3-phosphate; GAPDH: Glyceraldehyde 3-phosphate dehydrogenase; 1,3 BPG: 1,3-bisphosphoglycerate; PEP: Phosphoenolpyruvate; PK: Pyruvate Kinase; G6PD: Glucose-6-phosphate dehydrogenase; R5BP: Ribulose 1,5-bisphosphate; NADPH: Nicotinamide adenine dinucleotide phosphate; Acetyl CoA: Acetyl coenzyme A; ACLY: ATP citrate lyase; ACC: Acetyl-CoA carboxylase; FAS: Fatty acid synthase; HMGACR: HMG-CoA reductase; IDH: Isocitrate dehydrogenase; SDH: Succinate dehydrogenase; FH: Fumarase; MDH: Malate dehydrogenase; OAA: Oxaloacetate; CS: Citrate Synthase; ACS: Acetyl CoA Synthase; FAO: Fatty Acid Oxidation; CPT: Carnitine palmitoyltransferase; GLUD: Glutamate dehydrogenase; GLS: Glutaminase, NADH: Nicotinamide adenine dinucleotide; FADH2: Flavin adenine dinucleotide, CoQ: Coenzyme Q; Cyt C: Cytochrome C; TCA: Tricarboxylic Acid Cycle. Image made in Biorender^TM^.

It has become increasingly evident that the immune system is tightly regulated by the metabolic state of tissues, and responds accordingly via modulation of metabolic events such as glycolysis, the tricarboxylic acid (TCA) cycle, oxidative phosphorylation (OxPhos), and amino acid metabolism (Figure 2) [36]. Activated immune cells need faster energy production, so they upregulate their metabolic pathways (Figure 2) to meet the demand for various anabolic processes e.g., proinflammatory signalling via cytokine secretion. For instance, activated CD4+ helper T cells and CD8+ effector T cells have significantly increased glycolytic activity, resulting in a rise in intracellular and extracellular pH levels due to elevated lactate and CO_2_ emission from these cells, which allows for their proliferation and clonal expansion [37,38]. Cells can also induce alterations in their TCA cycle activity (e.g., accumulation of certain metabolites such as citrate and succinate in proinflammatory M1-like macrophages [39]), increase glutaminolysis [40] and upregulate the Pentose Phosphate Pathway (PPP) and fatty acid synthesis (FAS). These metabolic shifts in activated cells are observed both in adaptive immune cells, like effector T and B cells, as well as in innate immune cells like M1-like macrophages or natural killer (NK) cells [36,41]. On the contrary, pathways like fatty acid oxidation (FAO) allows for increased mitochondrial respiration via oxidative phosphorylation (OxPhos), a key metabolic pathway for Adenosine triphosphate (ATP) generation in quiescent and anti-inflammatory immune cells, e.g., M2-like macrophages and T_reg_ cells [42].

Concerning immunomodulation, exercise has long been known as an influencer of metabolism and immunity [26]. A long-term immune-related benefit of regular exercise, especially when combined with healthy diet, is reduced systemic inflammation, a condition that underlies most common chronic disorders [43,44]. For instance, a study found intensive, whole-body exercises like running and cycling caused a significant shift in various lipid super-pathway metabolites in human plasma, with a distinct fatty acid oxidation (FAO) signature [45,46], a process known to be upregulated in anti-inflammatory immune cells e.g., M2-like macrophages and T_reg_ cells. Exercise was also shown to downregulate surface expression of toll-like receptors (TLRs) in monocytes and macrophages, which in turn diminished their downstream inflammatory cascades [47,48,49]. Likewise, exercise has also been described to promote the switching of M1-like inflammatory macrophages to an anti-inflammatory M2-like phenotype in mice, and reduced the infiltration of macrophages into adipose tissue, resulting in a reduction in the secretion of proinflammatory cytokines and adipokines [50,51].

Comprehensive reviews describing links between immune phenotype and metabolism by specific innate and adaptive immune cells have been published recently [42,52]. There the authors describe in detail how defective metabolic signaling impacts upon immune cell function, and thus modulating metabolism in immunity possesses potential for therapy in diseases like cancer and autoimmunity. In the following sections of the review, we aim to amalgamate exercise immunology research with immunometabolism findings, both in the context of MS specifically. We will sequentially discuss some of the putative metabolic pathways and modulators dysregulated in MS, examine the literature on whether exercise is shown to modulate these pathways, especially in the immune system, and what that entails for the potential treatment of MS.

### 2.2. Metabolic Pathways and Modulators Disrupted in MS

#### 2.2.1. Glycolysis (Glucose Metabolism)

**Figure 3:**
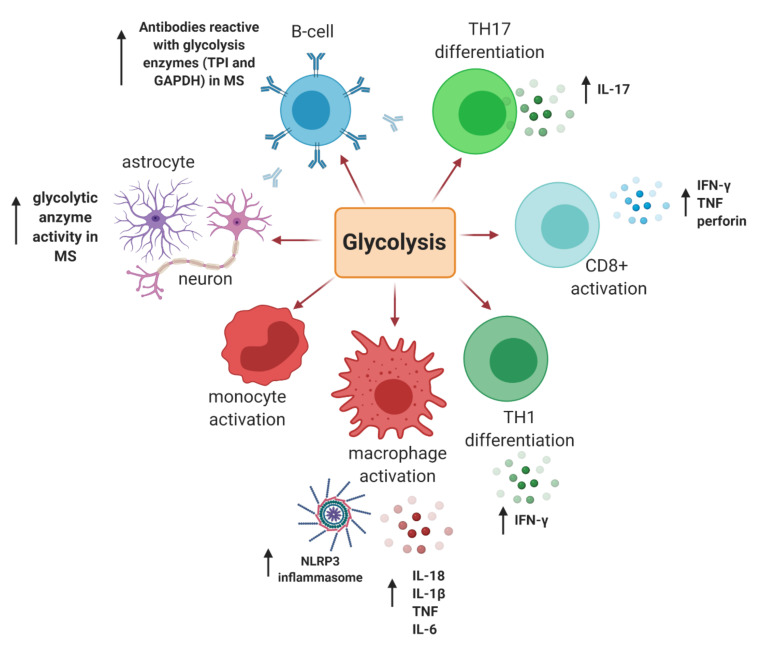
**A summary of the impact glycolysis has on cells in MS:** Glycolysis is a major pathway via which glucose is metabolised and generally is involved in activation of various immune cell subsets that are pathological in MS. Activation of immune cells such as in monocytes, macrophages and CD8+ T cells, and differentiation of CD4+ T-cell subsets, such as T_H_1 and T_H_17, leads to production of pro-inflammatory cytokines and cytotoxic granules. IL: Interleukin; TNF: Tumor Necrosis Factor; IFN: Interferon; TH: T Helper Cell; TP1: Topoisomerase 1; GAPDH: Glyceraldehyde 3-phosphate dehydrogenase; MS: Multiple Sclerosis. Image made in Biorender^TM^.

The immune system predominantly uses glucose, glutamine, and fatty acids as their main energetic substrates [53]. Glucose, however, is the main substrate for immune cell stimulation and is the first metabolite utilised for energy production [36]. Studies have established that monocytes and macrophages largely use glucose for their activation, and inhibition of glycolysis, a key pathway via which glucose is metabolised (Figure 2), results in suppression of in vitro macrophage activation and concomitant inflammation [54,55]. Moreover, the glycolytic enzyme, hexokinase 1 (HK1), is an important stimulator of the NLR family pyrin domain containing 3 (NLRP3) inflammasome, which stimulates the secretion of proinflammatory interleukin 1β (IL-1β) and interleukin 18 (IL-18), and induces cell death via pyroptosis [56]. In effector T cells, glycolysis allows for the increased secretion of interferon-γ (IFNγ) by CD4+ T cells, as well as the activation of cytotoxic CD8+ T cells. It also results in hyper-activation of the nutrient sensor mammalian target of Rapamycin (mTOR) as well as Hypoxia Inducible Factor 1 (HIF-1) pathways, which reprogram T cells to a more proinflammatory T_H_17 phenotype (Figure 3) [57].

Research describing general abnormal glucose metabolism in MS date as far back as the 1960s. The first group observed elevated blood pyruvate levels in both fasting and postprandial times in individuals with RRMS compared to healthy volunteers [58]. Another study showed glycolytic enzymes like enolase, pyruvate kinase, lactate dehydrogenase (LDH), and aldolase activity levels were increased in the CSF of individuals with disseminated sclerosis [59]. A different study looking at astrocytes and neuronal axons in active MS lesions demonstrated increased expression of various enzymes involved in glucose metabolism (Figure 3) [60]. Likewise, increased glycolysis was also detected in the inflammatory lesions in the CNS of EAE mice. [61]. The presence of glucose in the bloodstream also triggers the production and release of insulin, which increases the rate of transport of glucose from the blood into other cells of the body, such as muscles and fibroblasts. Interestingly, insulin resistance is reported to be frequent in individuals with MS [62,63]. Furthermore, it was reported that individuals with MS had an increased rate of impaired glucose tolerance (i.e. abnormally increased levels of glucose in blood) and their rate of lipid oxidation was reduced [64].

Impaired glycolysis is also evident in various immune cell subsets in MS: B cells and antibodies reactive with glycolysis enzymes triose phosphate isomerase (TPI) and Glyceraldehyde 3-phosphate dehydrogenase (GAPDH) were shown to be present intrathecally in CSF and MS lesions (Figure 3) [65]. Impairments in T-cell glycolytic activity were reported in relapse-remitting MS (RRMS) patients compared to healthy controls [66,67]. On the other hand, Kaushik et al. showed that macrophages transmigrating from perivascular cuffs into the CNS parenchyma are highly glycolytic. These cells were shown to express prominent levels of monocarboxylate transporter-4 (MCT-4), a transporter specialized in secreting lactate from glycolytic cells. Inhibition of this transporter in EAE mice resulted in diminished leukocyte infiltration as well as reduced overall disease severity. Relevance to MS was validated by the strong expression of MCT-4 and LDH in perivascular inflammatory macrophages in brains of MS patients [68]. Most recently, a study showed that the MS immunomodulatory drug Dimethyl Fumarate (DMF) inhibits the glycolysis enzyme GAPDH in activated myeloid and lymphoid cells, both in vitro and in vivo in mice and humans [69], showing inhibition of abnormal glycolysis and glucose metabolism might be one way to modulate pathology in activated immune cells in MS.

To that end, exercise has shown to improve glucose metabolism in lifestyle-related diseases like diabetes and obesity [70]. During exercise, glucose gets heavily oxidized in order to maintain heart rate and muscle contractions, resulting in a decrease of this substrate in the bloodstream, and hence, leading to less availability for immune cells, which then rely on oxidation of lipids instead [71]. Performing a single bout of exercise after a few days on a low carbohydrate (low glucose) diet resulted in an immunosuppressive profile characterized by diminished T cell, NK cell, and neutrophil function [72]. Exercise was also shown to prevent age-associated decreases in insulin sensitivity, and this was in part attributable to an increase in the insulin-sensitive glucose transporter, GLUT4, in rat skeletal muscle [70]. Furthermore, only twelve weeks of high-intensity aerobic exercise combined with some resistance training was successful at improving glucose tolerance significantly in MS, as patients showed significantly lower plasma glucose levels post-exercise [73].

#### 2.2.2. Fatty Acid Metabolism

**Figure 4:**
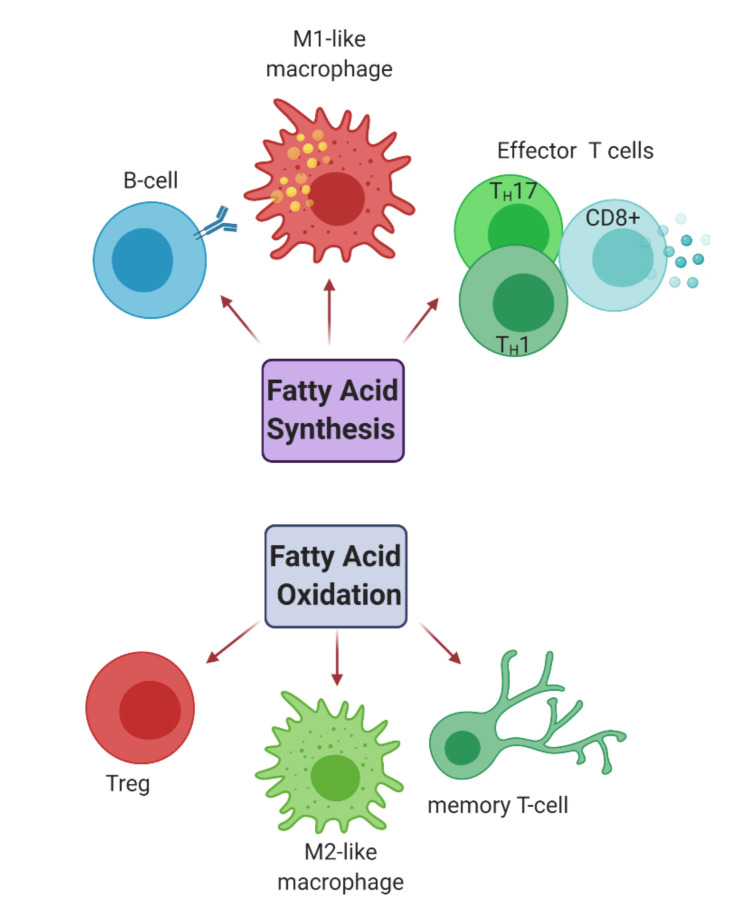
**Fatty Acid metabolism in immune cell subtypes:** Fatty acid oxidation (FAO) is primarily utilised by immunoregulatory cell types such as anti-inflammatory M2-like macrophages and T_reg_ cells to boost oxidative respiration by their mitochondria, whereas synthesis of fatty acids and cholesterol is increased in effector T-cells and B-cells for differentiation and development as well as in M1-like proinflammatory macrophages. Image made in Biorender^TM^.

**Figure 5:**
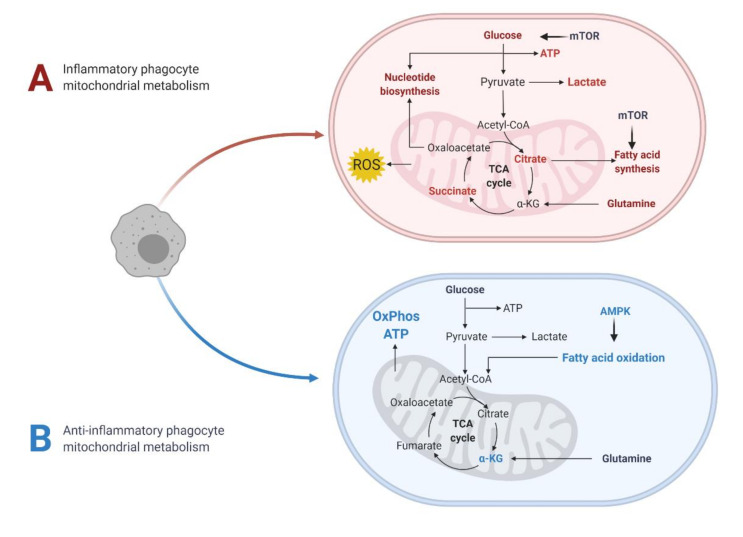
**Mitochondrial metabolism in determining macrophage phenotype:** Inflammatory macrophages constitute one of the largest immune cell infiltrates in CNS in MS. Proinflammatory macrophages have a distinct metabolic profile where they have reduced ATP production via OxPhos, altered TCA cycle metabolites (such as enhanced citrate and succinate levels), and have upregulated glycolysis and fatty acid synthesis pathways via mammalian target of Rapamycin (mTOR) activation. Such dysregulated mitochondrial metabolism allows these cells to be highly bactericidal by secreting inflammatory cytokines and ROS. On the other hands, immunoregulatory macrophages rely predominantly on TCA cycle and electron transport chain mediated OxPhos to generate ATP. This is aided by increased FAO via 5’ AMP-activated protein kinase (AMPK) activation. Image adapted from Biorender^TM^.

The activation of immune cells and the cytokines secreted by them, which affect transcriptional changes in the neighbouring cells, is strongly associated with fatty acid metabolism. For instance, M2-like macrophages, activated memory T cells, and T_regs_ have their lipid uptake increased that they then channel toward re-esterification and β-oxidation (also termed FAO) (Figure 4). This oxidation requires import of fatty acyl-CoA groups into the mitochondria from the cytosol. However, the outer mitochondrial membrane is impermeable to such molecules, and so they require shuttling through the membrane by conjugating with the compound L-carnitine as acylcarnitines. An enzyme called CPT-1 (Figure 2) catalyses this rate-limiting step in FAO. Conversely, proinflammatory phagocytes like M1-like macrophages incline to store excess fatty acids like triacylglycerols and cholesteryl esters as lipid droplets. In these cells, the synthesis of fatty acids is an important anabolic pathway that occurs in the cytosol and begins with the rate-limiting carboxylation of acetyl-CoA to malonyl-CoA via acetyl CoA carboxylases (ACCs). One function of malonyl-CoA is to inhibit fatty acids from conjugating with carnitine via regulation of CPT-1. By this means, it can prevent fatty acids from entering the mitochondria and undergoing FAO. Fatty acid synthesis (FAS) is enhanced in these cells via upregulation of different pathways: Firstly, due to an altered TCA cycle in these cells, excess mitochondrial citrate is exported to the cytosol where it is converted to acetyl-CoA, the main substrate for FAS. This is necessary to allow for membrane expansion, which is crucial for antigen presentation. Secondly, mTOR activation also upregulates FAS and nicotinamide adenine dinucleotide phosphate (NADPH) from the Pentose Phosphate Pathway (PPP) is used as a co-factor for *de novo* FAS, which favours the expansion of the endoplasmic reticulum (ER) and Golgi to support inflammatory cytokine secretion (See Figure 5). Other immune cells subtypes, such as effector CD4+ and CD8+ T cells as well as B cells, also rely on FAS for their development and differentiation (Figure 4) [41,53,74,75].

Given the abundance of fatty acids in the CNS, mounting evidence points towards importance of regulating fatty acid metabolism in MS [74]. For example, cholesterol (generated from FAS) plays essential roles in biological processes like cell membrane stability and myelin formation [76]. However, downstream metabolites of cholesterol termed oxysterols are heavily associated with MS pathology. The primary oxysterols, synthesized directly from cholesterol, are comprised of side-chain oxysterols and ring-modified oxysterols. Side-chain oxysterols include 24S-, 25-, (25R)-, 26- and 27-hydroxycholesterol (-OHC), and ring-modified oxysterols include 7α- and 7β-OHC and 7-ketocholesterol (-KC) [77]. A study reported levels of 24S-OHC were significantly lower in *advanced* primary progressive MS (PPMS) and older RRMS patients [78]. Contrastingly, increased 24S-OHC blood levels were reported in early neurodegenerative processes in MS [79]. Interestingly, the disease-modifying therapy natalizumab, is shown to reduce the levels of 24S-OHC in the CSF [80]. It has been hence proposed that a decrease of 24S-OHC after natalizumab treatment might reflect reduced neuronal damage [77]. Additionally, examination of CSF of MS patients found elevated concentrations of another oxysterol, 7-KC, which is known to stimulate neuronal damage via the activation and migration of microglia [81]. *In vitro*, expression and secretion of IL-1β was found to be induced by 25-OHC, and to a lesser extent by 27-OHC, in human macrophages [82].

Hence, modulation of fatty acid metabolism could assist in downregulation of inflammation in the CNS and be a potential therapeutic strategy in MS. As an example, soraphen A, an inhibitor of acetyl-CoA carboxylase 1 (ACC1), the enzyme that catalyses the conversion of acetyl-CoA to malonyl-CoA, was able to ameliorate EAE by downregulating IL-17 and IFNγ producing CD4+ T cells [83,84]. This corroborated with findings in EAE where acetyl L-carnitine supplementation provided antioxidant and anti-apoptotic effects in brain and spinal cord tissue when compared to the untreated EAE group [85]. Additionally, L-carnitine supplementation reportedly ameliorated fatigue in MS patients [86]. Previous in vitro and in vivo studies in mice have also shown that L-carnitine decreases the number of CD4+ and CD8+ T cells in the spleen, events that correlated with the reduction of serum IL-2 [87]. Furthermore, a case-study patient presenting with a relapsing and remitting demyelinating disorder similar to MS was found to have defective mitochondrial FAO and accumulated acylcarnitines in her blood, providing indirect evidence for impaired FAO in MS [88]. Similarly, inhibition of HMG-CoA reductase, that catalyses conversion of HMG-CoA to mevalonate prior to cholesterol synthesis (Figure 2), was shown to prevent paralysis in EAE mice via blockade of *de novo* FAS in T_H_1 and T_H_17 cells, and concomitant promotion of T_H_2 responses involving IL-4 and TGF-β secretion [89]. Likewise, deletion of 25-hydroxylase, responsible for 25-OHC synthesis, significantly attenuated disease severity by restricting the entry of T lymphocytes into the CNS [90].

Exercise acutely increases FAO, and endurance training has shown to increase the capacity to oxidize fat in skeletal muscle [91]. This is achieved via increased mitochondrial biogenesis, increased FAO enzyme activity, and enhanced capacity for oxygen delivery to muscle. Research also suggests that endurance exercise increases mRNA and protein expression of fatty acid transporters, which aid in the uptake of fatty acids by the mitochondria for FAO [92,93]. Moreover, exercise induces a state of energy deficit (lower ATP:AMP ratio) thereby causing AMPK activation (*See* Section 2.2.5 *mTOR and AMPK: nutrient sensing pathways*). Activation of AMPK stimulates FAO primarily through phosphorylation and inactivation of its downstream target acetyl-CoA carboxylase (ACC), the enzyme that synthesizes malonyl CoA [94,95]. Unsurprisingly, whole-body prolonged exercises resulted in post-exercise increases in FAO products, and decreases in plasma triacylglycerol esters and phospholipids have been observed in athletes [96,97]. Most recently, a study also showed low-intensity exercise was able to improve mitochondrial oxidative bioenergetics and increased FAO in peripheral blood mononuclear cells (PBMCs) in sedentary individuals [98].

In the context of exercise in MS and fatty acid metabolism, a study highlighted significant abnormality in the lipolytic response to endurance exercise in MS patients, who were shown to be less capable of utilising FAO during an exercise bout [99]. Considering in other diseases like obesity and type 2 diabetes, regular endurance exercise has shown promise in terms of improvements in FAO capacity as well as better glycaemic control [100], it would make sense to prescribe similar exercise regimes to improve FAO capacity for MS. This could be inferred further from a study that showed 12 weeks of medium-intensity training resulted in MS patients already displaying an improved blood cholesterol profile (reduced low-density lipoprotein) [101]. However, future studies addressing the link between increased FAO and anti-inflammatory immune polarization specifically in MS with exercise training are required to determine a more direct link between MS, exercise and fatty acid immunometabolism.

#### 2.2.3. Mitochondrial Oxidative Stress

Mitochondria are highly dynamic and adaptable to a cell’s energy requirements. Not only are they the site of the TCA cycle and amino acid metabolism, they are the hub for the electron transport chain (ETC) and OxPhos that generates most of the energy a cell needs in the form of ATP (Figure 2). Alterations in mitochondrial metabolic pathways (TCA cycle, ETC, and FAO) can lead to entirely different outcomes for immune cell function [102]. For example, inflammatory macrophages, which infiltrate the CNS in MS and EAE, exhibit an altered TCA cycle and downregulated OxPhos, allowing them to perform their proinflammatory roles by rerouting their biosynthetic resources towards the production of bactericidal products. By contrast, anti-inflammatory or tissue reparative macrophages have an intact TCA cycle and OxPhos (Figure 5) [103]. To illustrate the importance of mitochondrial metabolism further from a clinical perspective, T cells from RRMS patients have reduced mitochondrial oxidative respiration, which was reversible upon IFNβ treatment [66].

In addition to their impact on immune cell function, general mitochondrial respiration aberrations have been observed in MS patients. For example, defects in a component of Complex I of the ETC in white matter lesions have been reported [104]. Moreover, abnormal functional activity of complexes I and III, as well as reduced ATP synthase gene expression have been described in the motor cortex of MS patients [105]. Likewise, early mitochondrial dysfunction is evident in EAE as the mitochondria presented with a swollen morphology even in normal appearing white matter (NAWM) [106]. Comparable changes were observed in post-mortem MS tissue, suggesting that similar mitochondrial processes play a role in active MS lesions [107]. Interestingly, analysis of mitochondrial enzymes in muscle showed that in MS, muscle fibres of all types had lower succinate dehydrogenase (SDH: component of complex II of the ETC) activity, suggesting that muscles in individuals with MS rely more on anaerobic than aerobic oxidative ATP production compared to muscles of healthy individuals [108]. Taken together, these studies show mitochondrial respiratory chain abnormalities potentially cause functional disturbances in the demyelinated axons, muscles and immune cells in MS.

A better understanding of the molecular pathways leading to mitochondrial respiratory dysregulation could open up new therapeutic targets to combat neurodegeneration in MS. Mitochondria are one of the main sites in the cell for the production of reactive oxygen and nitrogen species (ROS/RNS), which directly inhibit the respiratory chain complexes, driving enhanced secretion of inflammatory cytokines from activated immune cells [40,103]. However, balance of ROS is critical, as an excess can lead to a vicious cycle of cellular damage [105,109]. Even though there is debate on whether ROS are a cause or consequence of pathology, there are many studies showing a link between ROS/RNS and pathological damage. For instance, ROS are shown to result in injury to intercellular tight junctions of endothelial cells thereby rupturing the selective permeability of the BBB. This facilitates trans-endothelial migration of monocytes into the brain’s parenchyma, where they differentiate into proinflammatory macrophages [110]. Similarly, loss of mitochondrial respiratory function by ROS/RNS-induced oxidative damage to membrane phospholipids and mitochondrial DNA can result in reduced ATP production that, if left uncompensated, leads to a loss of tissue function and, consequently, clinical symptoms such as muscle fatigue in MS and EAE [111,112].

A number of transcription factors, including nuclear respiratory factors (Nrfs), oestrogen-related receptor α, and peroxisome proliferator-activated receptors (PPARs), tightly coordinate expression of key OxPhos genes and antioxidant response genes to combat excessive ROS production [113]. Accordingly, a decrease in a transcription factor complex containing Nrf2 in MS grey matter samples has been observed, that correlated with decreased expression of electron transport chain subunit genes and increased oxidative damage [114]. Similarly, a significant decrease in PGC-1α in normal appearing grey matter (NAGM) samples was identified in progressive MS cases [115]. Treatment with antioxidant enzymes, such as catalase or superoxide dismutase 2 (Sod2), in animal models of MS resulted in a gradual reduction in the demyelination process, decreased trans-endothelial monocyte migration, and increased protection against endothelial cell death [116,117,118]. Notably, the immunomodulatory drug DMF used as a therapy for RRMS, is a potent activator of the Nrf2 antioxidant pathway [119].

Exercise performed in aged mice showed significant restorative effects on brain mitochondrial function through its impact on the ETC [120]. Similarly, regular high-intensity treadmill running has been shown to result in increased mitochondrial biogenesis in 21-month-old mice [27,121]. Certainly, it is well established that regular aerobic exercise results in increased activity of antioxidant enzymes in different organs [113]. In line with this, five weeks of treadmill training has been shown to increase the antioxidant response in exercised animals. This was shown to be mediated by the activation of skeletal muscle PGC-1α which, besides its crucial role in mitochondrial biogenesis, is also involved in the activation of the key antioxidant enzyme Sod2 [122]. Additionally, EAE mice that were subjected to endurance exercise training exhibited decreased levels of cytotoxic nitric oxide in the CNS, and had restored Nrf2 protein levels compared to non-exercised animals [23]. In this regard, the ability of physical exercise to dampen ROS production and modulate the redox status could limit their deleterious effects in neurodegeneration.

#### 2.2.4. Glutamine/Glutamate

Glutamine is a key substrate that provides intermediates for many metabolic reactions. Glutaminolysis is a prominent energy-producing process in immune cells, such as T_H_17 cells and proinflammatory macrophages (Figure 2). Activated T cells for instance, incubated without glutamine cannot proliferate or produce IL-2 or IFNγ [123]. Moreover, glutamine anaplerosis, a mechanism of regenerating TCA cycle metabolites that are used up for biosynthetic reactions, is increased in M1-like proinflammatory macrophages [53]. Glutamate, a key neurotransmitter in the CNS, is also derived from glutamine—which is taken up by cells via the amino acid transporter, Slc1a5—through the activity of the enzyme glutaminase (Figure 2) [71]. Dendritic cells (DCs), monocytes and macrophages have been shown to release glutamate after an inflammatory stimulus. This leads to increased migration and proliferation of autoreactive T cells. T cells can then induce the release of glutamate from antigen presenting cells, which is speculated to result in a positive feedback loop [53,124]. The abnormal secretion of glutamate by immune cells is toxic for neurons and oligodendrocytes, as it increases intracellular calcium, which causes mitochondrial damage via release of reactive oxygen species (ROS) and autophagic activity disruption [125]. Defective mitochondria accumulate within axons, rendering them sensitive to energy deficits and therefore, prone to degeneration [126].

People with primary or secondary progressive MS have increased levels of glutaminase in areas of nerve fibre damage in white matter lesions [127]. Moreover, glutamine has previously been reported to be significantly increased in the plasma of MS patients compared with healthy controls. It has also been shown that glutamate excitotoxicity and build-up plays a major role in MS by destroying myelin-producing oligodendrocytes [128,129]. The glutamate antiporter (via which glutamate is released by activated immune cells) is upregulated in peripheral blood leucocytes, optic nerve, and spinal cord samples from MS patients, as well as in spinal cord samples from EAE mice. For this reason, both glutamate and glutamine are suggested to be used as biomarkers of MS pathology [128]. Moreover, the glutamatergic receptor antagonist Memantine was efficacious in treating acquired fixational pendular nystagmus (oscillations of the eyes disrupting the visual acuity) in patients with MS [130], which has led to a phase 4 clinical trial (NCT02466191). In EAE, glutamine transporter Slc1a5-deficient mice have been shown to display less severe clinical scores when compared with their respective littermate controls, evidenced by the presence of fewer lesions and lesser number of infiltrating immune cells in the CNS. Furthermore, in vitro experiments showed an inherent impairment of Slc1a5-deficient naïve CD4+ T cells in differentiating into T_H_1 or T_H_17 phenotypes, whereas development into the T_reg_ subtype was not affected [123,124]. In fact, restricting glutamine promoted human T_reg_ cells with high FOXP3 expression [131].

Considering the above facts, one way via which exercise can aid immunomodulation in MS could be by regulating glutamine and glutamate levels. It has been suggested that skeletal muscle, that synthesizes most of the glutamine, plays an integral part in proper immune function [124,132]. Accordingly, both the plasma concentration of glutamine and immune-cell activation were reportedly decreased after prolonged endurance exercise [71,132,133,134]. Another study suggested that exercise induced an increase in mitochondrial glutamate metabolism through changes in the malic-aspartate shuttle (MAS) [135]. MAS utilizes the reversible reactions of malate to oxaloacetate to aspartate to “shuttle” cytosolic NADH produced during glycolysis into the mitochondria. This reaction is aided by the conversion of glutamate to alpha-ketogluterate. Overall, it results in increased ATP production by transference of the reducing equivalent NADH into complex I of the ETC. Hence, exercise-induced momentary increases in glutamate uptake and metabolism by the mitochondria may offer a mechanism for the delayed progression of neurodegenerative diseases (which generally have impaired glutamate metabolism) [136].

#### 2.2.5. mTOR and AMPK: Nutrient Sensing Pathways

mTOR is a major signalling kinase that impacts on various aspects of cellular function, comprising metabolism, development and proliferation. mTOR responds to both extracellular signals, such as hormones and growth factors (insulin, insulin-like growth factor-1), pattern recognition receptors, cytokines, as well as intracellular cues like nutrient abundance and cellular energy changes (AMP:ATP ratio) [137]. Concerning the immune system, suppressed mTORC1 activation in a mouse model has been associated with low macrophage numbers in bone marrow, impaired monocyte-to-macrophage differentiation, and an inhibition of their phagocytosis ability [138]. Some studies also showed that mTOR signalling contributed to the activation of brain microglia [18,139]. Similarly, others showed that mTOR signalling was increased in microglia (CD11/OX42+) in response to oxidative stress, as well as cytokines TNF and IL-1β, and this resulted in the production of the cytotoxic molecule, nitric oxide (NO) [109,140]. Additionally, the mTOR pathway was shown to be crucial for B-cell development and function. In fact, the mTORC1 inhibitor, rapamycin, could suppress human B-cell proliferation, reducing the number of antibody-producing cells [141,142]. Other studies observed that mTOR activation is crucial for proper migration of CD8+ T lymphocytes [143], and for cell cycle and proliferation of CD4+ T cells [144].

In the context of MS, it has been shown in RRMS patients that T_reg_ cell proliferation post-stimulation is diminished, owing to hyper-activation of S6 ribosomal protein (a target of mTOR) [9]. Selective knock-out of the positive regulator of mTOR, Rheb, in T cells resulted in reduced leukocyte infiltration in the spinal cord and decreased clonal expansion of T_H_1 and T_H_17 cells in the periphery, overall leading to a milder disease score in EAE [144]. Blocking mTOR activity itself has proven beneficial in rats, where mTOR inhibition by rapamycin significantly ameliorated EAE progression by augmenting the T_reg_ proliferative capacity [145]. Such data delivers insight into how mTOR inhibition can result in immunomodulation [143,146].

Just like mTOR, adenosine monophosphate kinase (AMPK) is also a nutrient sensor that works to maintain the energy homeostasis of cells. A decline in ATP levels of the cell activates AMPK that then downregulates anabolic processes, like protein synthesis. AMPK and mTOR play contrasting roles in cells as mTOR inhibition is critical for AMPK-dependent metabolic homeostasis [147]. This was demonstrated in T cells by examining AMPK-deficient T cells, which presented with increased glycolytic activity, elevated phosphorylation of mTORC1 targets, and increased secretion of IFN-γ upon antigen receptor stimulation. Furthermore, the last two effects were reversed by addition of rapamycin. Moreover, sustained AMPK activation was shown to reprogram T cells to restrict glucose oxidation and lipid synthesis, and instead favoured FAO [94]. Finally, loss of AMPK was shown to result in an exacerbated EAE disease course, with profound infiltration of mononuclear cells compared to wild-type mice. Spleen cells isolated from those AMPK-deficient EAE mice also exhibited a significant increase in IFNγ levels [148].

It is notable that insulin and leptin, two hormones secreted in over-fed states, inhibit the AMPK pathway. Conversely, in a starvation or fasted state, the AMPK pathway is activated via hormones like adiponectin and ghrelin. AMPK activation then induces expression of transcriptional regulator peroxisome proliferator-activated receptor-*γ* coactivator 1*α* (PGC-1*α*) in skeletal muscle, which controls mitochondrial biogenesis. Moreover, through PGC-1*α* phosphorylation, aerobic exercise influences other transcription factors, like peroxisome proliferator-activated receptor-*γ* (PPAR*γ*), as well as estrogen-related receptors, ERR*α* and ERR*γ*, which control mitochondrial energy metabolism via the tricarboxylic acid (TCA) cycle, FAO, and oxidative phosphorylation [149,150]. Endurance and, to a lesser extent, resistance exercises have been shown to activate AMPK, and inhibit mTOR in non-muscular tissues as well, such as liver and fat [151]. This leads us to surmise that this metabolic setting also works synergistically with the immune system, which has its proinflammatory phenotype aggravated in states of overfeeding and nutrient excess, triggering chronic low-grade inflammation, and in states of fasting and exercise, an anti-inflammatory state.

It is important to note however, the differential role of nutrient sensors such as mTOR in different tissue and cell types. For instance, mTOR activation mediates neural network remodelling, synaptic transmission and myelination. Moreover, it is also important for the attainment and maintenance of memory paradigms [152]. The integral role played by mTOR in the myelination process and neuronal plasticity can hence limit the use of general mTOR inhibitors, and may require specific timing and cell-specific targeting to avoid interference with processes of remyelination and axonal regeneration that occur after initial damage. For instance, on a translational level, phase II clinical trials of the rapamycin analogue CCl-779 (Temsirolimus) for RRMS were completed but failed to progress to phase III (NCT00228397) [153,154]. In the future, it will be advantageous to test the impact of exercise specifically in such immune cell nutrient sensing pathways in MS patients.

#### 2.2.6. Tryptophan/Kynurenine

Another key metabolic modulator that has been subject of research in autoimmunity is the amino acid Tryptophan (Trp) and its derivatives. Mammals consume Trp for protein synthesis, the release of immunomodulatory catabolites, the synthesis of the neurotransmitter serotonin, as well as the hormone melatonin to name a few [155]. Certainly, Trp metabolism can occur through four distinct pathways, the most significant being the kynurenine (Kyn) pathway. This is because metabolites of the Kyn pathway are known to influence the vascular system, immune tolerance, and infections. Trp degradation by the enzyme indoleamine-2,3-dioxygenase (IDO) is a rate-limiting step for metabolic immune regulation, whose expression is increased by IFN-γ, TNF, and TLR stimulation [156]. Furthermore, Trp is one of the sources for NAD^+^ biosynthesis, an important coenzyme for metabolic processes controlling immune cell function, such as glycolysis, TCA cycle, and OxPhos [157]. 

Primary observations showed that Kyn metabolites, particularly Kyn itself, suppressed NK cell activity [158] as well as that of DCs, monocytes, and macrophages in mice [157]. Moreover, Kyn was shown to block T cell proliferation and even induced apoptosis in those cells [159]. However, IDO-mediated Kyn production in DCs led to the proliferation of the T_reg_ phenotype [155,160]. Employing the pharmacological inhibitor of IDO (by using 1-methyl-tryptophan) resulted in EAE exacerbation, suggesting a protective role of IDO metabolites in the disease [161]. Consistent with those results, estrogen administration led to IDO activation in DCs, with associated apoptosis of T cells, and concomitant decline in EAE severity. Hence, IDO activation was put forth as a mechanism via which estrogen mediated EAE suppression, and was proposed to be, at least partly, an explanation for the decreased rate of relapses observed in MS cases during pregnancy [162]. Apart from EAE findings, both the expression and activity of IDO was also significantly reduced in PBMCs isolated from RRMS patients when compared to healthy controls [163]. Furthermore, Kynurenic Acid (KynA), a downstream product of Kyn pathway, had been shown to reduce bacterial lipopolysaccharide induced TNF secretion from cultured PBMCs [164]. Interestingly as well, an increased Kyn/Trp ratio was detected in MS patients treated with IFN-β, a well-known therapy for RRMS, linking IDO induction as a possible mechanism of action for IFN-β [165].

It appears that activation of the kynurenine pathway results in two very distinct events depending on the activation duration. Short-term benefits include immunomodulation and neuroprotection whereas chronic activation of Kyn metabolising enzymes results in an accumulation of neurotoxic products (e.g., Quinolinic acid) which impede the remyelination process [166]. From a clinical standpoint, identifying the risk of developing a more progressive form of MS is vital. In this context, Lim et al. found that Kyn metabolite levels are a significant indicator of MS disease course: Changes in levels of Kynurenic acid (KynA) in the CSF occurred between RRMS patients in relapse versus recovery, as well as between RRMS and SPMS patients [167,168,169]. Furthermore, individuals with SPMS and RRMS also revealed different responses of Trp metabolism to acute aerobic exercise and training: In contrast to RRMS, the training interventions did not change Trp and Kyn/Trp ratio in persons with SPMS [167].

One consistent finding in the literature is that KynA is elevated in the circulation following acute endurance exercise [155,170,171]. Moreover, exogenous KynA administration equivalent to physiological “exercised” levels was enough to induce expression of anti-inflammatory cytokines in adipose tissue of mice that were on a high-fat diet [172]. This was revealed to be due to subsequent activation of PGC-1α, which together with PPARα/δ, was shown to shift peripheral Kyn metabolism toward the production of KynA [170,171]. Moreover, mice with transgenic expression of PGC-1α in skeletal muscle (and hence, higher levels of Kyn-metabolising enzymes) were shown to be resilient towards developing depressive-like behaviour [155,173]. Modulating Trp-Kyn metabolism via PGC-1α-PPAR activation in skeletal muscle could therefore become a new therapeutic preserving the immunosuppressant action of kynurenines via KynA, and concomitantly decreasing the neurotoxic effects associated with Kyn in chronic inflammatory conditions. Table 1 summarises how exercise targets some of the key metabolic pathways discussed in this review compared to known pharmacological agents used in treating MS.

## 3. Conclusions

**Figure 6:**
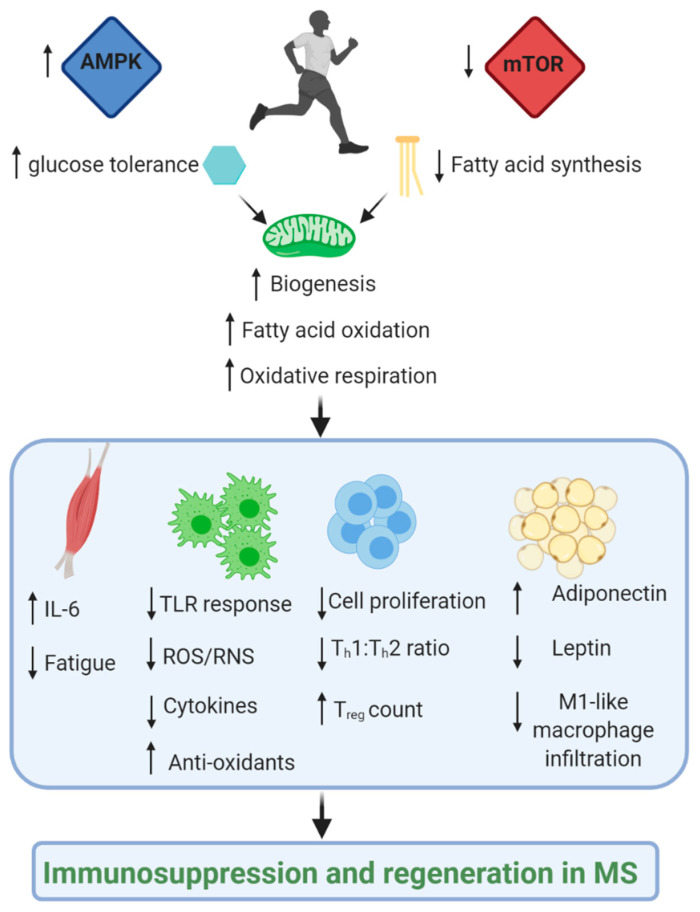
**Impact of exercise on immune-inflammatory response and metabolic function.** Regular aerobic/endurance exercise has shown to induce a tolerogenic glucose state, which is associated with a state of immunomodulation through changes in amino acid metabolism (e.g., increased kyunrenic acid and glutamate oxidation) and nutrient sensor pathways (increased AMPK:mTOR ratio). This induces increases mitochondrial biogenesis and oxidative respiration at the mitochondria at least partly through increased fatty acid oxidation (FAO) and decreased fatty acid synthesis (FAS). This impacts on various organs and cells, including (1) higher myokine IL-6 secretion by skeletal muscle tissue; (2) Decreased proinflammatory macrophage and effector T cell number and cytotoxic activity; (3) Decreased ROS and proinflammatory cytokine production by innate immune cells, as well as an increased antioxidant response; (4) increase in T_reg_ number and function; (5) decrease in leptin secretion (likely due to reduced fat mass) and increase in adiponectin secretion, and lower proinflammatory M1-like macrophage infiltration. Image made in Biorender^TM^.

Exercise can reprogram metabolism in immune cells by secretion of cytokines, modulation of cellular energy sensors and immunometabolic regulators (Figure 6). Mechanistic knowledge in these areas could lead to clinical trials using specific exercises (potentially with nutritional or pharmaceutical therapies (see Table 1)) to target specific metabolic pathways in immune cells to enhance or suppress their functions, as required. To this end, further studies will be needed to decipher the metabolic aberrancies occurring in pathogenic immune cells of patients with MS compared to healthy controls, and how these changes associate with disease progression and exercise therapy.

## 4. Limitations

Studies have shown that MS often leads to an inactive lifestyle due to difficulties in engaging in physical activity, especially as the disease progresses [174,175]. This inactivity accelerates the physical deconditioning process in a vicious cycle. It needs to be deduced then if the muscular and metabolic anomalies (e.g., obesity) observed in MS are a secondary symptom due to this physical inactivity or a primary cause of the disorder. Furthermore, the heterogeneity of MS makes research on the effects of exercise difficult. For instance, exercise that may be effective in a person with ataxia may not be appropriate for patients whose primary symptom is sensory loss [176]. Moreover, there is disparity between what constitutes a specific form of exercise making it difficult to compare data across various studies. Additionally, studies devoted solely to patients with progressive forms of MS are limited, and even when patients with the progressive disease are included alongside those with RRMS, the number of patients sampled is usually small. Finally, not all studies take into account the impact of treatment patients are on, and how that can potentially impact the disease course with exercise.

## Figures and Tables

**Table 1:** Comparison of pharmacological therapies and exercise therapy in targeting metabolic pathways altered in MS.

Metabolic Pathway	Pharmacological Drugs			Exercise Therapy	
	Drug	Effect	REF(S)	Effect	REF(S)
**Glycolysis**	DMF	↓ Glycolysis Enzyme GAPDH In Myeloid and Lymphoid Cells	[69]	↓ Glycolysis ↑ Glucose Tolerance	[70,72,73,174]
**Fatty Acid Metabolism**	Natalizumab	↓ Inflammatory Oxysterol 24S-OH In CSF	[80]	↑ FAO ↓ Cholesterol LDL Synthesis	[92,95,97,98]
**Mitochondrial Oxidative Stress**	DMF	↓ Ros ↑ Nrf2 Antioxidant Response Pathway	[175]	↓ Ros ↑ Pgc1α ↑ Antioxidant Response	[22,113,121,122]
**Glutamate**	Memantine	↓ Glutamate Excitotoxicity	[130]	↓ Glutamate by Increasing Glutamate Uptake and Metabolism	[136,176]
**mTOR/AMPK**	Temsirolimus	↓ mTOR Activity	[154]	↓ mTOR ↑ AMPK	[95,151]
**Kynurenine**	IFN-β	↑ Kyn/Trp Ratio	[165]	↑ KynA (due to ↑ PGC-1α)	[155,171]
